# A scalable model for EPA and fatty acid production by *Phaeodactylum tricornutum*

**DOI:** 10.3389/fbioe.2022.1011570

**Published:** 2022-10-12

**Authors:** Wenjia Gu, John M. Kavanagh, Dale D. McClure

**Affiliations:** ^1^ School of Chemical and Biomolecular Engineering, The University of Sydney, Darlington, NSW, Australia; ^2^ Department of Chemical Engineering, College of Engineering, Design and Physical Sciences, Brunel University, London, United Kingdom

**Keywords:** microalgae, modelling, fatty acid, EPA, photobioreactor, phaeodactylum tricornutum

## Abstract

Large-scale photoautotrophic production of microalgae has the potential to provide a sustainable supply of omega-3 fatty acids (eicosapentaenoic acid (EPA) and docosahexaenoic acid (DHA)) for human and animal nutrition. This study presents a kinetic model for the EPA-producing microalga *Phaeodactylum tricornutum* in photoautotrophic conditions, with light and nitrogen being the growth limiting factors. The model was developed using a dataset obtained from bench-scale (5 L) cultures and was successfully validated against pilot-scale (50 L) cultures. This model is the first to predict the biomass and total fatty acid accumulation along with the EPA concentrations in the biomass and total fatty acid fraction for microalgae. The model was used to develop an optimized repeated-batch strategy; implementation of this led to increases in the biomass and EPA productivities of 50 and 20% respectively. This clearly indicates the potential of the model to be used as a tool in the design, optimization and scale-up of microalgal systems for EPA production.

## Introduction

Eicosapentaenoic acid (EPA) is an omega-3 fatty acid which is essential for human and animal nutrition (Glencross, 2009; Tocher, 2010; Hamilton et al., 2020). The majority of EPA is produced from wild-caught fish, however meeting the global demand for EPA in this way is becoming increasingly challenging due to sustainability concerns (Hamilton et al., 2020). These factors have driven increased interest in alternative methods of EPA production. One method which has been commercialized is the production of EPA using heterotrophic microorganisms, these can be either thraustochytrids (Barclay et al., 2010), or engineered yeasts (Xie et al., 2015). An alternative route is photoautotrophic production using microalgae, which are the primary producers in marine food chains (Hamilton et al., 2020).

Processes using heterotrophic microorganisms have the advantage of much higher biomass productivities than photoautotrophic processes (Barclay et al., 2010), resulting in more favourable process economics. While photoautotrophic processes are less productive they may offer advantages from a sustainability perspective. For example, photoautotrophic production does not need potable water or arable land, and hence will not compete with food production. This contrasts with processes based on heterotrophic growth which rely on carbohydrate feedstocks (Barclay et al., 2010; Xie et al., 2015). The use of wild type organisms and sustainable production technologies may also mean that photoautotrophic production technologies have a favourable consumer perception. As the large-scale, photoautotrophic production of omega-3 fatty acids is currently limited by the process economics (Borowitzka, 2013; Gu et al., 2021) there is a clear need for the identification of suitable strains and the development and optimization of scalable, cost-effective production processes.

The marine alga *Phaeodactylum tricornutum* (Bacillariophyta) has been identified as a promising candidate for large-scale, photoautotrophic production of EPA. Its EPA content was amongst the highest reported for wild-type organisms (3–5% of its dry cell weight) (Gu et al., 2021). Its relatively high growth rate and good robustness make it suitable for both indoor and outdoor cultivation (Butler et al., 2022; Gu et al., 2022). Despite its potential, *P. tricornutum* remains a minor product in the algal industry; in Europe, its annual production volume was estimated to be merely 4 tonnes, 2.8% of that of *Spirulina* (Cyanobacteria) (Araújo et al., 2021). Hence, there is a clear need to develop scalable cultivation processes for *P. tricornutum* in order to achieve large-scale photoautotrophic EPA production. It is increasingly being recognized (Noorman and Heijnen, 2017; Crater and Lievense, 2018) that the development of reliable, accurate models is extremely useful in the successful scale-up of biotechnological processes. In order to facilitate process scale-up and optimization, any model developed should be applicable at a range of scales and operating conditions. Similarly, it must be capable of providing reliable predictions of key operating parameters (e.g., the biomass and EPA concentrations).

Several models have been proposed for the growth of *P. tricornutum*. Given that light is one of the most important factors that governs the growth of phototrophic organisms, most models included light as a growth limiting factor (Acién Fernández et al., 1998; Acién Fernández et al., 2001; Bitaubé Pérez et al., 2008; Dillschneider and Posten, 2013; San Pedro et al., 2013; Ozcan and Ovez, 2020). The typical approach was formulating the specific growth rate as a Monod-type function of the average light intensity in the culture (Acién Fernández et al., 1998; Acién Fernández et al., 2001; Bitaubé Pérez et al., 2008; San Pedro et al., 2013). Apart from light, nutrients (e.g., nitrogen and phosphorus) (Dillschneider and Posten, 2013; Coppens et al., 2014) and temperature (Bitaubé Pérez et al., 2008; Ozcan and Ovez, 2020) have also been included as factors which influence the growth. Among the proposed models, only Dillschneider and Posten (2013) considered the accumulation of lipid and carbohydrate along with biomass production. The authors proposed a linear programming approach that optimized the distribution of energy (ATP, NADPH) and key nutrients (N, P, S) between the functional biomass and the intracellular storage compounds (lipids, carbohydrates) with the objective of maximizing the productivity of the total biomass. These existing models provided good predictions for their proposed application; however, no existing model has attempted to incorporate EPA accumulation for *P. tricornutum*.

The composition and concentration of fatty acids in microalgae can be affected by the growth conditions (Breuer et al., 2012). For *P. tricornutum*, limitations in key nutrients such as nitrogen and phosphorous can trigger accumulation of triacylglycerides (TAGs), these can be up to 30% of the dry cell weight (Breuer et al., 2012; Remmers et al., 2017). This is a universal phenomenon observed in *P. tricornutum* strains of different geographical origins (Abida et al., 2015). The *de novo* synthesis of palmitic acid (C16:0) and palmitoleic acid (C16:1n7) is responsible for the vast majority (∼90%) of the increase in the fatty acid content (Breuer et al., 2012; Abida et al., 2015). Meanwhile, EPA is transferred from membrane lipids to TAG, but its *de novo* synthesis is not significant; the net result is that the specific EPA concentration (on a biomass basis) remains approximately constant, while the percentage of EPA as a fraction of the total fatty acids decreases due to the synthesis of shorter chain fatty acids (Breuer et al., 2012; Remmers et al., 2017; Huang et al., 2019). Environmental factors including light, salinity and temperature, were shown to have little effect on the specific EPA concentration of *P. tricornutum* (Remmers et al., 2017; Gu et al., 2022). From the perspective of process development and scale-up, such behaviours are significant for two reasons. First, the fact that the specific EPA concentration is not strongly affected by growth conditions means that process optimization should focus on maximizing the biomass productivity of *P. tricornutum*. Secondly, as it may be desirable to fractionate the algal biomass to produce an oil product it is important to understand both the total fatty acid concentration as well as the fraction of EPA in the total fatty acids. Therefore, a model that can predict the biomass and EPA production as well as the EPA fraction in the total fatty acids will be a useful tool in process development and scale-up.

Therefore, the aim of this study is to develop and validate a model for the production of EPA by *P. tricornutum*. In particular this work aims to: 1) develop a model which can simultaneously predict the biomass, EPA and fatty acid concentrations; 2) develop a model which can be applied to a range of reactor designs, scales and operating conditions and 3) use the model to optimize the cultivation process.

## Materials and methods

### Algal strain and stock cultures

*Phaeodactylum tricornutum* CS-29 was obtained from the Australian National Algal Culture Collection. Stock cultures were maintained in 25 ml *f*/2 medium in 50 ml Erlenmeyer flasks. *f*/2 medium was prepared at a salinity of 35 g L^−1^ using a commercially available marine salts mixture. The composition of *f*/2 medium was NaNO_3_ (880 μM), NaH_2_PO_4_ (36 μM), NaSiO_3_ (140 μM), FeCl_3_ (12 μM), CuSO_4_ (41 nM), ZnSO_4_ (76 nM), Na_2_MoO_4_ (37 nM), CoCl_2_ (37 nM), MnSO_4_ (940 nM), disodium EDTA (12 μM), thiamine hydrochloride (300 nM), biotin (2 nM) and cyanocobalamin (400 pM). The flasks were placed on a cool-white LED pad with a light intensity of ∼60 μmol m^−2^ s^−1^ and a photoperiod (light:dark) of 12:12 h. The cultures were not aerated, but manually agitated approximately every 2 days, and were maintained at 20–25°C. Stock cultures were transferred into fresh medium every 3–4 weeks.

### Experimental set-ups

The flat-panel PBRs in this work were based on the design described previously (McClure et al., 2018). The PBRs had a working volume of 5 L and a light path length of 0.05 m. Cultures were performed in batch mode and were inoculated with 150 ml inoculum that had been grown in flasks for approximately 1 week, giving an initial optical density of ∼0.05 (measured at a wavelength of 550 nm). The growth medium was prepared using deionized water and a commercial marine salt mix (Quantum^®^ Mixed Macro Probiotic Salt™, Quantum Aqua, Australia). Inorganic nutrients were supplemented at four times of the *f*/2 strength (denoted as 4 × *f*/2).

Temperature was maintained at 21–23°C by circulating chilled water through a stainless-steel coil. Air enriched with 1% (v/v) CO_2_ was introduced at 5 L min^−1^ from the bottom through a sparger with evenly spaced 1 mm holes. The pH was checked using pH strips (MColorpHast™, Merck KGaA, Darmstadt, Germany) to ensure that cultures were not pH-inhibited or CO_2_-limited.

Light was provided unidirectionally by four cool-white-light LED bars (9 W, 6000 K color temperature, Jaycar, Australia) attached on the surface of the PBRs. The light was operated at a photoperiod of 12:12 h (light:dark). The light intensity was measured using a Walz ULM-500 light meter (Walz GmbH, Effeltrich, Germany). Measurements were made with the PBR filled with cell-free medium and aerated at 5 L min^−1^. The light intensity was measured at eleven evenly spaced points across the front face of the PBR at a height of 250 mm; with the measured profile being shown in Supplementary Figure S1. To determine the average light intensity a parabolic function was fitted to the measured points. This function was then integrated and divided by the width of the PBR (200 mm) to determine the average light intensity. This gave an average light intensity of approximately 350 μmol m^−2^ s^−1^ (see Supplementary Figure S1). This average value was used as the incident light intensity ( $I_{o}$ ) for the model.

Samples were collected on 10 days between Days 4 and 16 for biomass, nitrate and fatty acid analyses. Prior to sampling, the sampling port was flushed thoroughly with approximately 50 ml of culture broth. Then, a 150 ml aliquot (3% of the culture volume) was withdrawn. This was replaced by an equal volume of nutrient-free marine salt solution. Losses due to evaporation were made up with deionized water.

**FIGURE 1:**
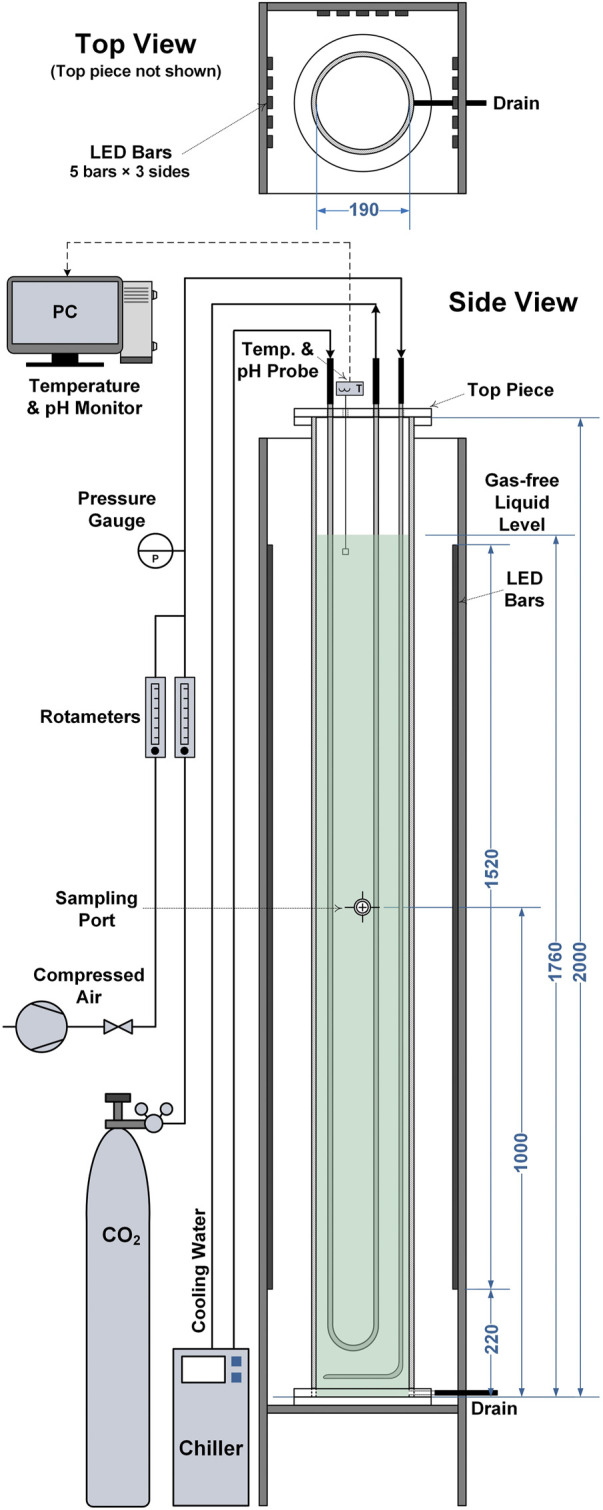
Schematic diagram showing the 50 L bubble column photobioreactors used in this study. All dimensions are given in millimetres.

To test the validity of the model against a wider range of reactor scales and designs cultivations were performed in 50 L bubble column PBRs. A schematic diagram of the bubble column PBRs is presented in Figure 1. The PBRs had a working volume of 50 L and a diameter of 0.19 m. Aeration was provided by introducing air containing 1% (v/v) CO_2_ at 20 L min^−1^ (0.4 vvm) through a stainless-steel sparger located at the base of the column. Light was provided from three sides of the PBRs by LED light banks at a photoperiod of 16:8 h (light:dark). Each light bank gave an incident light intensity of approximately 360 μmol m^−2^ s^−1^, the locations where this was measured are indicated in Figure 1. This gave a total light intensity of approximately 1,000 μmol m^−2^ s^−1^. The culture temperature was maintained at 21 ± 1°C by circulating coolant through a stainless-steel U-shaped cooling coil. The culture pH was monitored online using an InPro 3250i probe (Mettler-Toledo, Columbus, the United States). For all experiments it was observed that the pH was in the range of 7–8, indicating that the cultures were not limited by mass transfer of CO_2_.

The 50 L cultures were carried out in batch mode. The inocula were grown in the same 5 L flat-panel PBRs described above for 4–6 days, after which 2.5–4 L of broth was transferred into the 50 L PBRs to give an initial optical density of approximately 0.08 (measured at 550 nm).

To investigate nitrogen-limited and nitrogen-sufficient conditions, the growth medium was prepared at two strengths. Nitrogen-limited cultures were grown in medium with all nutrients prepared at 5 × *f*/2 concentrations (as this was predicted to lead to nitrogen becoming depleted between days 4–6); whereas for the nitrogen-sufficient cultures, nitrate and phosphate were boosted to 15 × *f*/2 concentrations while other nutrients were prepared at 5 × *f*/2 concentrations.

### Analytical methods

Biomass concentration was measured at regular time points. A known volume (typically 50–60 ml) of culture broth was collected and filtered through pre-weighed 0.55 μm filter paper (Advantec GA-55, Toyo Roshi Kaisha Ltd, Tokyo, Japan). The filter paper was rinsed with 150 ml 0.5 M ammonium bicarbonate solution to remove the salts, and then dried in an oven at 105°C overnight. Biomass productivity (mg L^−1^ day^−1^) was calculated as:

$$biomass\;productivity=\frac{DCW}{t}$$ (1)

where DCW is the dry cell weight (mg L^−1^) and t is the time (days) since Day 0.

The nitrate concentration in the growth medium was measured using a standard method (American Public Health Association, 2012) using a Cary 60 UV-Vis spectrophotometer (Agilent, Australia). Prior to analysis, the samples were filtered through 0.45 μm PTFE syringe filter (Advantec, Toyo Roshi Kaisha Ltd, Tokyo, Japan) to remove the cells. When necessary, the samples were diluted such that the absorbance at 220 nm was <0.8. The nitrogen concentration in the medium, N (mg L^−1^), was determined as:

$$N=\frac{14}{62}\frac{DF∙\left(ABS_{220}-2ABS_{275}\right)}{0.06007}$$ (2)

where *DF* is the dilution factor, ABS_220_ is the measured absorbance at 220 nm and ABS_275_ is the measured absorbance at 275 nm. The constant 0.06007 is a calibration factor relating the absorbance to the concentration of nitrate, and 14 and 62 are the molecular weights of nitrogen and nitrate, respectively.

The nitrogen cell quota, Q (mg N mg^−1^ DCW) for the Droop model was estimated as:

$$Q=\frac{N_{o}-N}{DCW}$$ (3)

where $N_{o}$ is the initial nitrogen concentration in the medium on Day 0 (mg L^−1^).

Analysis of the biomass absorption coefficient, $K_{a}$ (L mg^−1^ m^−1^), was based on the method by Romero Villegas et al. (2017). The samples were diluted such that the optical density was <0.8 and transferred to a cuvette with a path length of 10 mm. The same spectrophotometer was used to measure the optical density (OD) at 550 nm. The absorption coefficient was determined as:

$$K_{a}=\frac{ABS_{550}}{0.01∙DCW}$$ (4)

Microalgal cells were examined and imaged using a Nikon ECLIPSE Ci-L microscope equipped with a white LED illuminator. Bright-field images of unstained cells were captured at × 1,000 magnification using a digital camera.

Fatty acid concentrations were extracted using a modified form of the Bligh and Dyer method as described by Breuer et al. (2013). Briefly, water, chloroform and methanol were added to freeze dried biomass at ratios of 8, 10, and 20 µl per mg, respectively. Glyceryl triheptadecanoate was dissolved the chloroform (∼1.2 g L^−1^) and used as the internal standard. Samples were shaken and chloroform (20 µl per mg dry biomass) was added and the sample was mixed again, before being allowed to stand to facilitate phase separation. The chloroform layer was aspirated and the top layer was washed with chloroform (40 µl per mg dry biomass). The chloroform layers were then combined and the chloroform was evaporated. Transesterification of the lipids was done via the addition of 3 ml methanol containing 5% (v/v) concentrated sulfuric acid, samples were heated at 70°C for 3 hours with regular shaking. The lipids were extracted by the addition of 3 ml *n*-hexane and 3 ml water; 1 ml was aspirated from the hexane phase and used for the quantitation of the lipids using gas chromatography.

A Shimadzu GC2010 Plus system equipped with a FID detector was used to quantify the lipid concentrations, using a 30 m long FAMEWAX column (0.32 mm internal diameter, 0.25 µm film thickness) from Restek. An injection volume of 5 µl was used, the injection temperature was 250°C, the split ratio was 25:1 and helium was used as the carrier gas at a velocity of 0.25 m s^−1^. The oven temperature started at 130°C, increasing to 230°C over a period of 20 min. At the end of this time the oven temperature was maintained at 230°C for an additional 20 min. Fatty acids were identified using a reference FAME standard (Supelco 37 component FAME mix) which was purchased from Sigma Aldrich. Complete details of the method are available in Gu et al. (2022).

### Model development

The proposed model seeks to predict the light-and-nitrogen controlled growth of *P. tricornutum* as well as its total fatty acid and EPA concentrations. The model was formulated based on the following assumptions:

• The reactor is well-mixed, meaning that the concentrations of nutrients and dissolved CO_2_ are homogenous. This assumption is reasonable as the mixing time in the reactor is of the order of seconds, and is much smaller than the growth rate of the algae (in the order of hours). As the timescale for mixing is much smaller than that for nutrient uptake (which occurs on similar timescales to growth) it is very unlikely that significant gradients in nutrients will exist.
• It was assumed that the concentration of algae is homogenous in the reactor. This is a reasonable assumption, as the settling velocity of the algae (calculated using Stokes Law) is of the order 10^–6^ m s^−1^, much smaller than the liquid velocity likely to be found in the reactor (of the order 10^–2^ m s^−1^).
• Growth is not limited by the availability of carbon. Such a limitation would arise if the rate of mass transfer of carbon dioxide from the gas to liquid was not sufficient to compensate for the consumption of carbonate species by the algae. The assumption that the system is not limited by the mass transfer is justified by the fact that the pH remained in the range seven to nine for all experiments performed; if the rate of mass transfer was not sufficient the pH would increase due to the change in equilibrium between carbonate species
• An intrinsic assumption of the Droop model is that nitrogen uptake is a separate event from biomass growth.
• Growth inhibition due to high light intensities does not occur at the tested ranges. Terry’s work has reported that *P. tricornutum* could tolerate irradiance up to 1700 μmol m^−2^ s^−1^ without suffering photoinhibition (Terry, 1986). The incident light intensities used in this study (350 and 1,000 μmol m^−2^ s^−1^) were much lower than this value.
• Growth inhibition due to high nitrate concentrations was not considered.
• For the sake of simplicity, the algal biomass does not grow on internally stored carbon sources, meaning that the biomass growth ceases instantaneously when the dark phase begins.
• In this case we have assumed light attenuation only occurs in one dimension (i.e., along the light path). This choice was made to simplify the model, meaning Ordinary Differential Equations (ODEs) can be used in place of Partial Differential Equations (PDEs). In addition to considerably simplifying the model this assumption is reasonable given the experimental configurations examined.
• Light attenuation due to absorption and scattering is lumped into one parameter, the absorption coefficient ( $K_{a}$ ).
• The cell decay rate ( $K_{d}$ ) is not affected by the growth conditions and therefore is constant. This assumption is justified by the fact that in this study all experiments were carried out under well-regulated conditions with minimal variation in the temperature, salinity and pH.

The local light intensity, I (μmol m^−2^ s^−1^), at a given point in the culture is calculated using the well-established Beer-Lambert law:

$$I=I_{o}e^{-K_{a}Xl}$$ (5)

Where $I_{o}$ is the incident light intensity (μmol m^−2^ s^−1^); X is the biomass concentration (mg L^−1^); $K_{a}$ is the biomass absorption coefficient (L mg^−1^ m^−1^); l is the distance between the given point and the illuminated surface (m).

The average light intensity, $I_{av}$ (μmol m^−2^ s^−1^), experienced by the cells was determined by integrating the local light intensity along the light gradient and dividing the integral by the light path length. For the 5 L flat-panel PBRs, the average light intensity can be determined using the following expression:

$$I_{av}=\frac{I_{o}}{K_{a}XL}\left(1-e^{-K_{a}XL}\right)$$ (6)

where L is the depth of the 5 L flat-panel PBRs. The average light intensities at the beginning (Day 0) and the end (Day 16) of the batch cultures were predicted to be 343 μmol m^−2^ s^−1^ and 22 μmol m^−2^ s^−1^, respectively.

For the 50 L bubble column PBRs, light was supplied from three sides and light attenuates along both *x* and *z* directions (Figure 1), the average light intensity could be determined by solving the following expression:

$$I_{av}=\frac{I_{o}}{\pi R^{2}}\int_{0}^{R}\int_{0}^{2\pi }\left(e^{K_{a}Xl_{1}}+e^{K_{a}Xl_{2}}+e^{K_{a}Xl_{3}}\right)∙r∙d\theta ∙dr$$ (7)

where:

$$l_{1}=\sqrt{R^{2}-r^{2}\cos^{2}\theta }-rsin\theta$$ (8)

$$l_{2}=\sqrt{R^{2}-r^{2}\cos^{2}\theta }+rsin\theta$$ (9)

$$l_{3}=\sqrt{R^{2}-r^{2}\sin^{2}\theta }-rcos\theta$$ (10)

where R is the radius of the 50 L bubble column PBR, i.e. 0.095 m. The model predicted that the value of $I_{av}$ on Day 0 was approximately 1,000 μmol m^−2^ s^−1^, and on Day 14 the average light intensity was of the order 10–16 μmol m^−2^ s^−1^.

The cultures were described using three state variables: the biomass concentration ( X ), the concentration of nitrogen in the medium ( N ) and the nitrogen quota ( Q ) using the following set of ordinary differential equations (ODEs):

$$\frac{dX}{dt}=\mu X-K_{d}X$$ (11)

$$\frac{dQ}{dt}=U-\mu Q$$ (12)

$$\frac{dN}{dt}=-UX$$ (13)

where μ is the specific growth rate (day^−1^); $K_{d}$ is the biomass decay rate (day^−1^) and U is the uptake rate of nitrogen (mg N mg^−1^ DCW day^−1^).

The specific growth rate of algal biomass depends on the availability of both light and nitrogen. A Monod-type equation based on the approach used by San Pedro et al. (2013) was used to model the effect of the light intensity. The effect of nitrogen was accounted for using the Droop model, which decouples the algal growth and the nitrogen availability in the medium; instead, the growth rate is related to the intracellular nitrogen cell quota. It was assumed that the cells have a maximum capacity of storing nitrogen, i.e. $Q_{\max}$ , while growth ceases when Q reaches the minimal cell quota, $Q_{\min}$ (Flynn, 2008). The specific growth rate under the combined effect of light and nitrogen limitation was formulated as:

$$\mu =\mu_{\max}\left(\frac{I_{av}^{n}}{K_{I}^{n}+I_{av}^{n}}\right)\left(\frac{1-\frac{Q_{\min}}{Q}}{1-\frac{Q_{\min}}{Q_{\max}}}\right)$$ (14)

where $\mu_{\max}$ is the maximum growth rate (day^−1^); $K_{I}$ is the half-saturation constant of light (μmol m^−2^ s^−1^); n is an exponent (dimensionless).

The nitrogen uptake rate depends on both the external nitrogen concentration and the intracellular nitrogen cell quota (Bernard, 2011):

$$U=U_{\max}\left(\frac{N}{K_{N}+N}\right)\left(1-\frac{Q}{Q_{\max}}\right)$$ (15)

where $U_{\max}$ is the maximum nitrogen uptake rate (mg N mg^−1^ DCW day^−1^); $K_{N}$ is the half-saturation constant of nitrogen (mg N L^−1^).

## Results and discussion

### Parameter determination

Experiments were performed in the 5 L PBRs to generate sufficient data for parameter determination as well as model validation. In these experiments 4 × *f*/2 medium was used, the light intensity was 350 µmol photons m^−2^ s^−1^ and the reactors were illuminated for 12 h per day. Results from these experiments are shown in Figure 6. These experiments were performed in triplicate.

The ODEs of the state variables (Eq, (11)–(13)) have eight parameters: $\mu_{\max}$ , $K_{I}$ , n , $Q_{\min}$ , $Q_{\max}$ , $K_{N}$ , $U_{\max}$ and $K_{d}$ . To determine the parameter values, a multivariate multiple regression procedure was run using the *fminsearch* command in Matlab 2019b (MathWorks Inc.). The procedure minimized the normalized root-mean square error (NRMSE) between the predicted and experimental data for X , N and Q , based on the method used by Regueira et al. (2021). An initial attempt was made with the errors being unweighted; however, this tended to result in overprediction of the biomass concentration at the late growth phase for N-sufficient batch cultures. Thus, weighting factors were introduced to improve the model accuracy (Gu et al., 2014; Ofungwu, 2014). As cultures are more likely to be harvested towards the end of a batch, it is more important for the model to be able to accurately predict the final biomass during the later growth phase than the lag and early exponential phases. Therefore, the weighting factors was formulated such that more weight was given to datapoints that are later in the growth curves.

$$NRMSE=\sqrt{\frac{1}{p∙q∙m}\overset{p}{\underset{k=1}{\sum }}\overset{q}{\underset{j=1}{\sum }}\overset{m}{\underset{i=1}{\sum }}{w_{i,j}\left({\hat{Y}}_{k,j,i}-Y_{k,j,i}\right)}^{2}}$$ (16)

Y and $\hat{Y}$ are the predicted and observed values, respectively; p is the number of state variables (i.e. 3); q is the number of days where samples were collected for this regression procedure (i.e. 10 days); m is the number of biologically independent replicates (i.e., 3); w is the weight factor. For the biomass concentration, w on a given day is the mean of the three replicates:

$$w_{j}=\frac{1}{m}\overset{m}{\underset{i=1}{\sum }}Y_{j,i}$$ (17)

For the nitrogen cell quota, w is the reciprocal of the mean of the three replicates:

$$w_{j}=\frac{m}{\overset{m}{\underset{i=1}{\sum }}Y_{j,i}}$$ (18)

For the external nitrogen concentration, the errors are unweighted, i.e., w=1 .

The initial value of $\mu_{\max}$ was estimated based on the experimental data of *P. tricornutum* cultures grown at a light intensity of 210 μmol m^−2^ s^−1^ (McClure et al., 2018). Optical density measured at 550 nm during the exponential phase (between Day 1,2 and Day 4,5) was linearized and the slope of the linear regression line was taken as $\mu_{\max}$ (see Supplementary Figure S2). The initial values of $Q_{\max}$ and $Q_{\min}$ were estimated based on the measured Q (using Eq. 3) on the first and last sampling days (Days 4 and 16), respectively. These times were chosen as on Day 4 the medium still contained nitrogen meaning this time point is likely to provide a reasonable estimate of *Q*_max_. Similarly, Day 16 was chosen as by the time the nitrogen in the medium had been depleted and the cultures had entered the stationary phase, meaning that it would provide a reasonable value of *Q*_min_. The initial values of $K_{d}$ , $U_{\max}$ , $K_{N}$ , $K_{I}$ and n were estimated based on values reported in the literature (see Table 1). The regression procedure was run for 50 iterations; after approximately 35 iterations the value of NRMSE did was approximately constant (the NRMSE decreased by 1% between iterations 35 and 50). The initial and fitted values are presented in Table 1.

**TABLE 1:** Initial and fitted values of parameters for the growth and nitrogen uptake model.

Parameter	Unit	Initial value	References	Fitted value
$\mu_{\max}$	day^−1^	2	McClure et al. (2018)	2.4
$K_{d}$	day^−1^	0.01	Edmundson and Huesemann, (2015)	0.010
$Q_{\min}$	mg N mg^−1^ DCW	0.04	this work	0.034
$Q_{\max}$	mg N mg^−1^ DCW	0.13	this work	0.12
$U_{\max}$	mg N mg^−1^ DCW day^−1^	0.5	Deschênes and Wouwer, (2016)	0.59
$K_{N}$	mg N L^−1^	0.01	Deschênes and Wouwer, (2016)	0.010
$K_{I}$	μmol m^−2^ s^−1^	60	San Pedro et al. (2013)	50
n	-	1.9	San Pedro et al. (2013)	1.9

The fitted values shown in Table 1 were found to be in good agreement with experimental work. For example, the maximum specific growth rate of *P. tricornutum* was in the range of 1.5–2.7 day^−1^ (Terry et al., 1983; Fawley, 1984; Geider et al., 1985; Acién Fernández et al., 2001; Bitaubé Pérez et al., 2008; San Pedro et al., 2013). The value found here ( $\mu_{\max}$ = 2.4 day^−1^) was consistent with the literature. Likewise, the minimum and maximum nitrogen quota reported in the literature for photoautotrophic algae were in the ranges of 0.01–0.07 g N g^−1^ DCW and 0.09–0.10 g N g^−1^ DCW, respectively (Packer et al., 2011; Adesanya et al., 2014; Benavides et al., 2015; Deschênes and Wouwer, 2016). Values determined in this work were in reasonable agreement with the literature, with the fitted value of *Q*_max_ being slightly (20%) higher than the maximum reported value. Similarly, values of the other fitted parameters were found to be in good agreement with those previously published (see Table 1).

**FIGURE 2:**
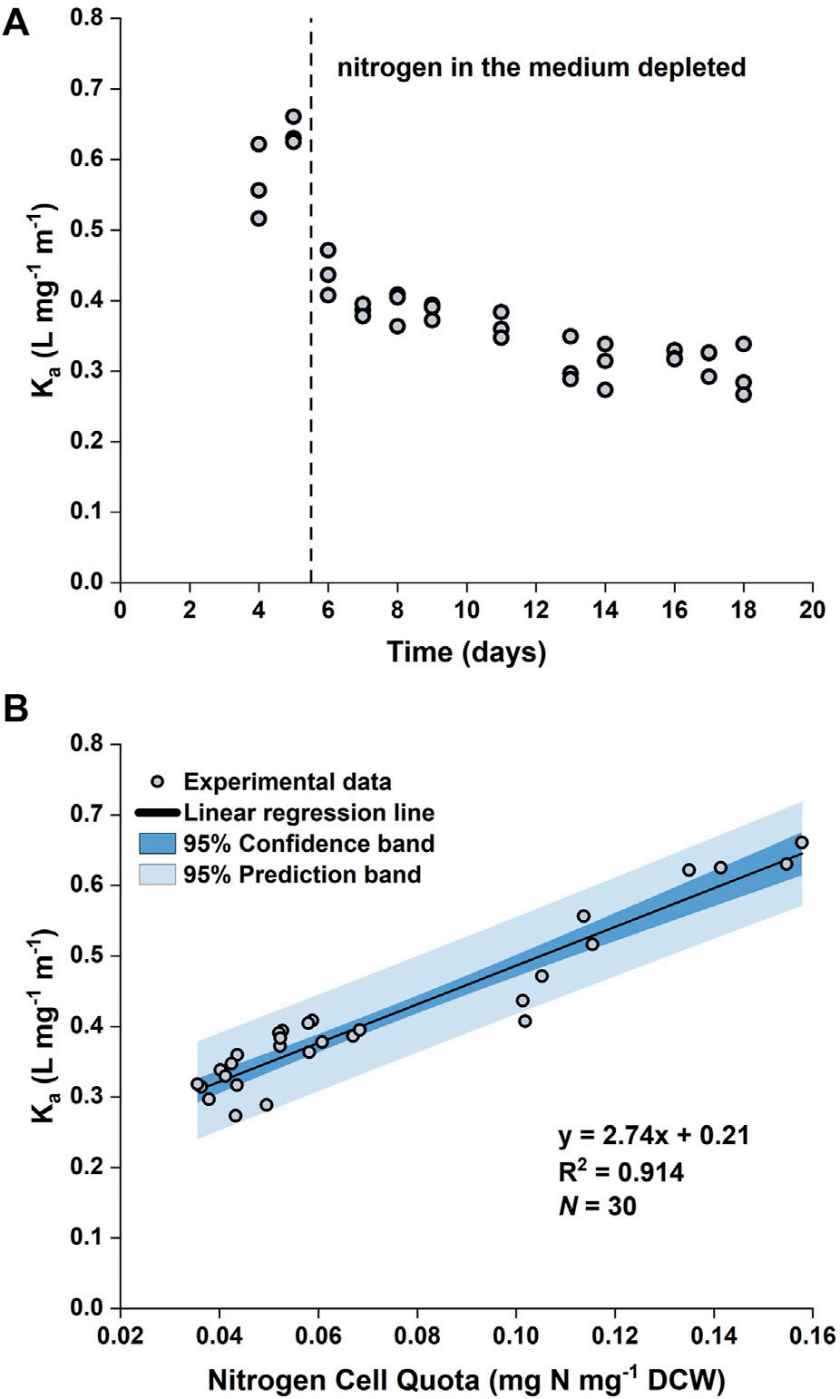
Time evolution of biomass absorption coefficient ( $K_{a}$ ) **(A)** and the relationship between $K_{a}$ and the nitrogen cell quota **(B)** in batch cultures of *P. tricornutum* under nitrogen-limited conditions. The batch cultures were grown in 5 L flat-panel PBRs with 4 × *f*/2 medium. Datapoints were pooled from three biologically independent replicates.

**FIGURE 3:**
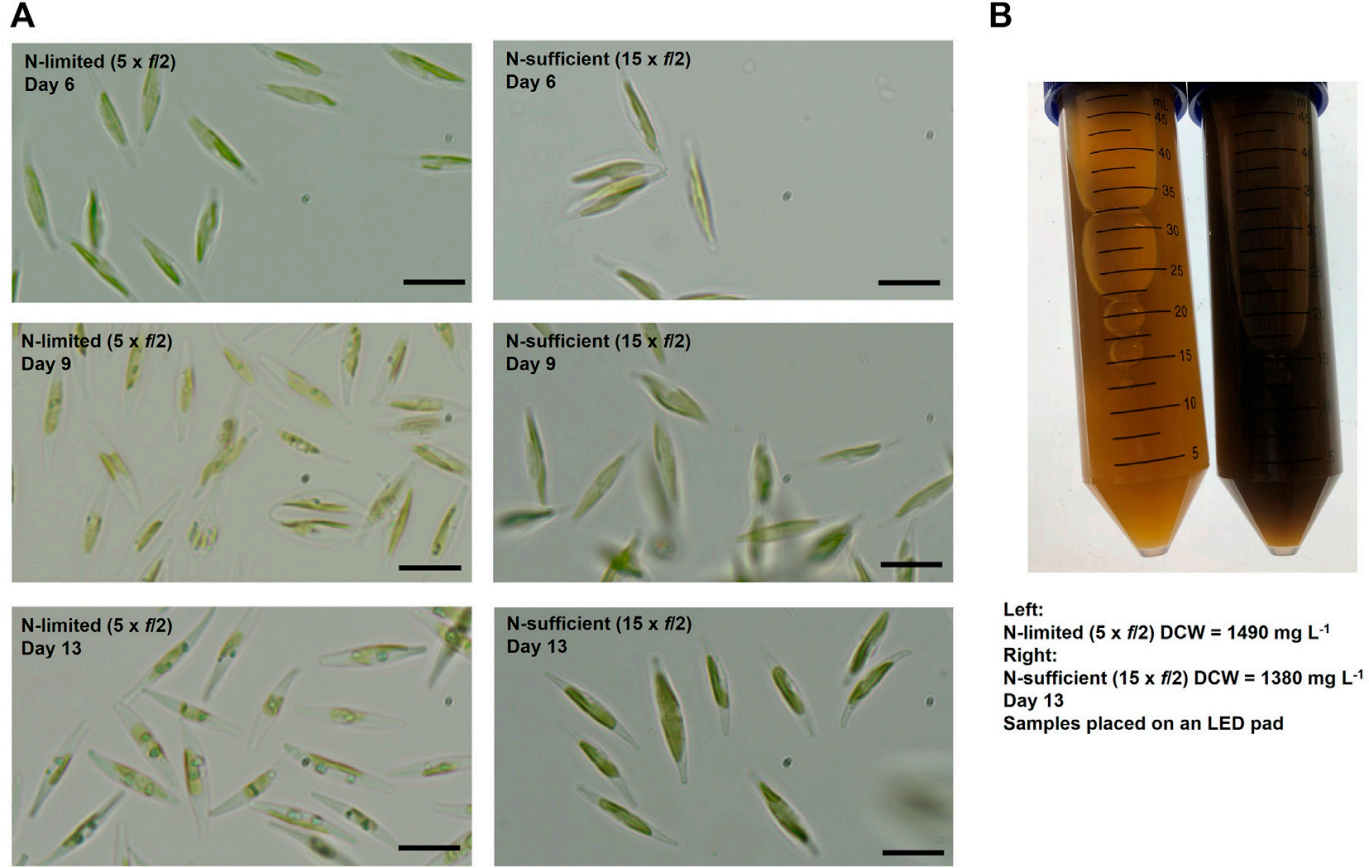
Changes in cell pigmentations over time **(A)** and comparison of samples collected on Day 13 **(B)** for batch cultures of *P. tricornutum* grown in nitrogen-limited (5 × *f*/2) and nitrogen-sufficient (15 × *f*/2) conditions. Scale bars represent 10 μm.

It was observed that the value of the biomass absorption coefficient ( $K_{a}$ ) changed over time (Figure 2A). The decrease in $K_{a}$ is mostly likely due to the loss of photosynthetic pigments as the cells redistribute nitrogen in response to its depletion in the growth medium (Levitan et al., 2015). Our previous work (McClure et al., 2018) has also shown that the specific concentration of fucoxanthin (a major carotenoid pigment in *P. tricornutum*) is higher under nitrogen replete conditions. Microscopic examination of the cells grown in the 50 L bubble column PBRs revealed that under nitrogen limitation there was an obvious difference in the pigmentation compared with that in nitrogen sufficient conditions (Figure 3A). The color of the nitrogen limited cultures was noticeably lighter and less opaque than that of the nitrogen sufficient cultures despite their similar biomass concentrations (Figure 3B). Longworth et al. (2016) reported that under nitrogen limitation the chlorophyll *a* content of *P. tricornutum* decreased by approximately 50%. This was in line with the decrease in $K_{a}$ value observed here (from 0.6 L mg^−1^ m^−1^ on Day 5 to 0.3 L mg^−1^ m^−1^ on Day 16).

By plotting $K_{a}$ as a function of Q , a linear relationship between $K_{a}$ and Q could be obtained (Figure 2B). Hence, a linear regression was performed using OriginPro 2019b (OriginLab), which gave the following relationship:

$$K_{a}=2.74Q+0.21$$ (19)

Eq. 19 was implemented in the model such that the value of *K*_*a*_ was a function of the nitrogen quota ( Q ).

**FIGURE 5:**
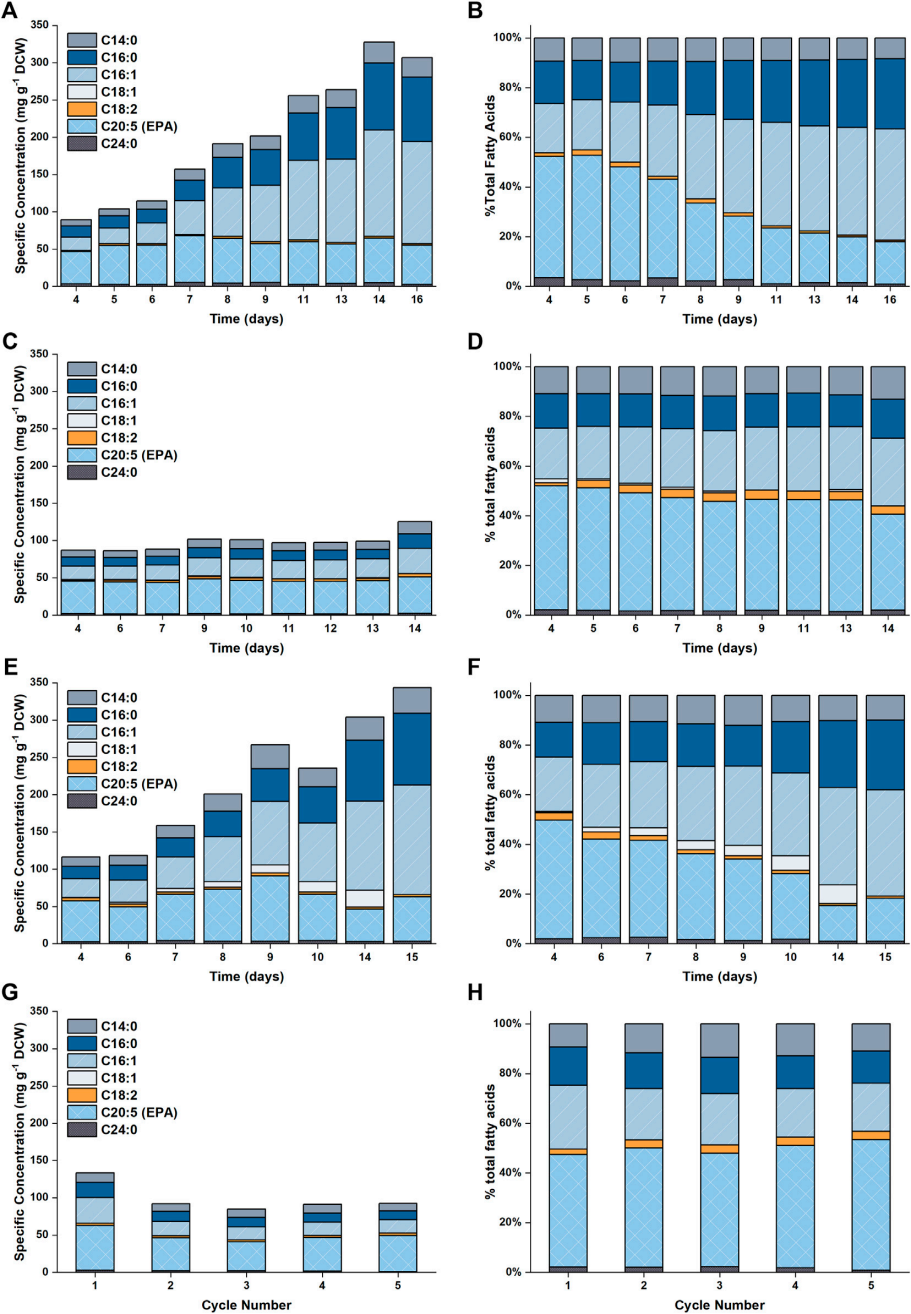
Plot showing the fatty acid profiles as a function of time for the different experimental conditions examined in this work. Results are shown in **(A,B)** for 5 L cultures **(C,D)** for 50 L cultures with N replete medium, **(E,F)** for 50 L cultures with N limited medium and **(G,H)** for the repeated batch cultivations.

The accumulation of lipids in *P. tricornutum* under nitrogen limitation has been widely reported in the literature (Breuer et al., 2012; Levitan et al., 2015; Longworth et al., 2016; Remmers et al., 2017; Remmers et al., 2018). Previous studies revealed that upon nitrogen limitation cell growth reduced and photosynthetically fixed carbon was channeled towards storage compounds, such as lipids, coupled with significant increase in the saturation level of these lipids (Levitan et al., 2015; Remmers et al., 2018). Here, it was observed that the specific concentration of total fatty acids (TFA) increased from ∼100 mg g^−1^ DCW to ∼300 mg g^−1^ DCW over the course of the batch (Figure 5 and Figure 6). This threefold increase in the TFA concentration was predominantly attributed to the accumulation of C16:0 and C16:1n7 fatty acids, and to a lesser extent, C14:0, which were shown to be the major fatty acid components in *P. tricornutum* (Lupette et al., 2019). The specific concentration of EPA did not vary significantly over the course of the batch; hence the net result was a progressive decrease in the EPA fraction in TFA over time.

By plotting the specific concentration of TFA, $C_{TFA}$ (mg g^−1^ DCW), as a function of Q , it was observed that the relationship could be described using a reciprocal function. Therefore, a nonlinear regression was performed using the Levenberg-Marquardt algorithm in OriginPro 2019, which gave the equation:

$$C_{TFA}=\frac{3.68}{Q^{1.32}}+42.2$$ (20)

**FIGURE 4:**
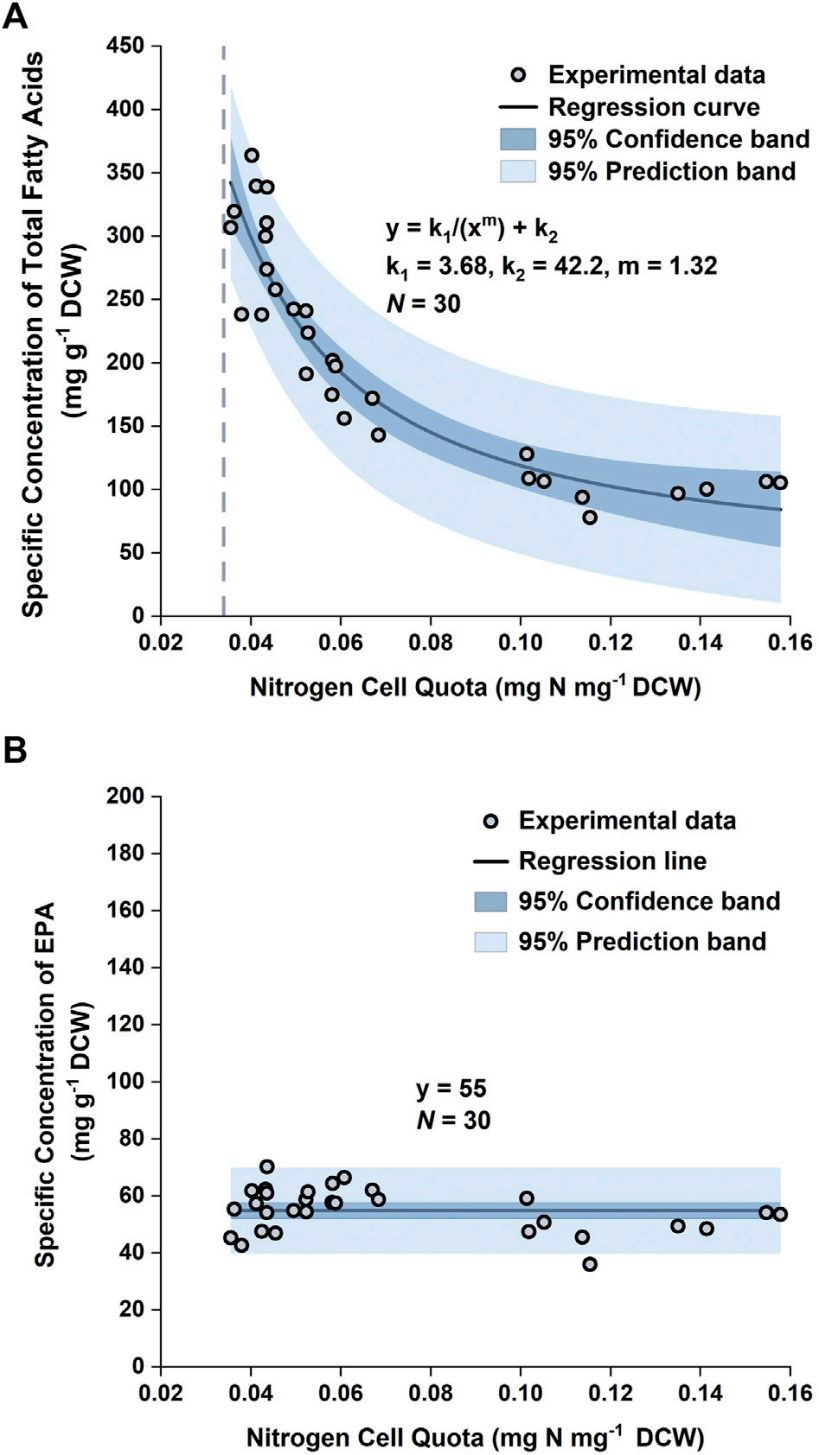
Specific concentration of total fatty acids in biomass versus nitrogen cell quota **(A)** and specific concentration of EPA in biomass versus nitrogen cell quota **(B)** in *P. tricornutum*. Datapoints were pooled from three biologically independent replicates.

As the EPA concentration in the biomass remained relatively unchanged, it could be approximated as a constant. Normality tests (Supplementary Figure S4) demonstrated that the datapoints of the specific concentration of EPA in the training set followed a normal distribution, with the mean and standard deviation being 55 mg g^−1^ and 7.7 mg g^−1^, respectively. Hence, the mean of the datapoints, 55 mg EPA g^−1^ DCW, was used as the specific concentration of EPA in the model (Figure 4).

### Sensitivity analysis

To examine the influence of each parameter on the simulation output and identify the most critical parameter(s), a sensitivity analysis was carried out using a one-at-a-time method each of the eight parameters listed in Table 1 was varied by ±20% while the other parameters were kept constant (Hamby, 1994). Here we have chosen to examine the effect on the biomass concentration and nitrogen cell quota, as these are the key variables for the system.

Under nitrogen sufficient conditions (i.e. $N\gg K_{N}$ ) the value of Q will approach $Q_{\max}$ _,_ meaning that the specific growth rate will depend on the values of $\mu_{\max}$ , $K_{I}$ and n . As shown in Supplementary Figure S3 and S6 this was found to be the case. As shown in Supplementary Figure S3 the nitrogen cell quota was almost solely governed by the value of $Q_{\max}$ under nitrogen replete conditions. Changing the values of $K_{N}$ , $U_{\max}$ and $Q_{\min}$ has virtually no effect on the system, with the resultant changes in the final biomass concentration and nitrogen cell quota being less than 0.4%.

Under nitrogen limited conditions Q approaches $Q_{\min}$ meaning that the specific growth rate (and hence the biomass concentration) is sensitive to the parameters for light and nitrogen uptake (see Supplementary Figure S7 and S8). In conditions where the nitrogen has been depleted from the medium growth is not affected by the values of $K_{N}$ and $U_{\max}$ .

To summarize, the model predictions are sensitive to the values of $K_{I}$ and $\mu_{\max}$ under both N-limited and N-sufficient conditions. Under nitrogen limited conditions the model predictions of the nitrogen cell quota are sensitive to the value of $Q_{\min}$ , while under nitrogen replete conditions the predictions are sensitive to the value of $Q_{\max}$ . Hence, it can be concluded that good predictions of the biomass production and EPA concentration requires accurate determination for $K_{I}$ , $\mu_{\max}$ , $Q_{\min}$ and $Q_{\max}$ . However, the model predictions were not sensitive to the values of $K_{d}$ , $K_{N}$ and $U_{\max}$ in any of the scenarios tested.

### Validation for 5 L PBRs

**FIGURE 6:**
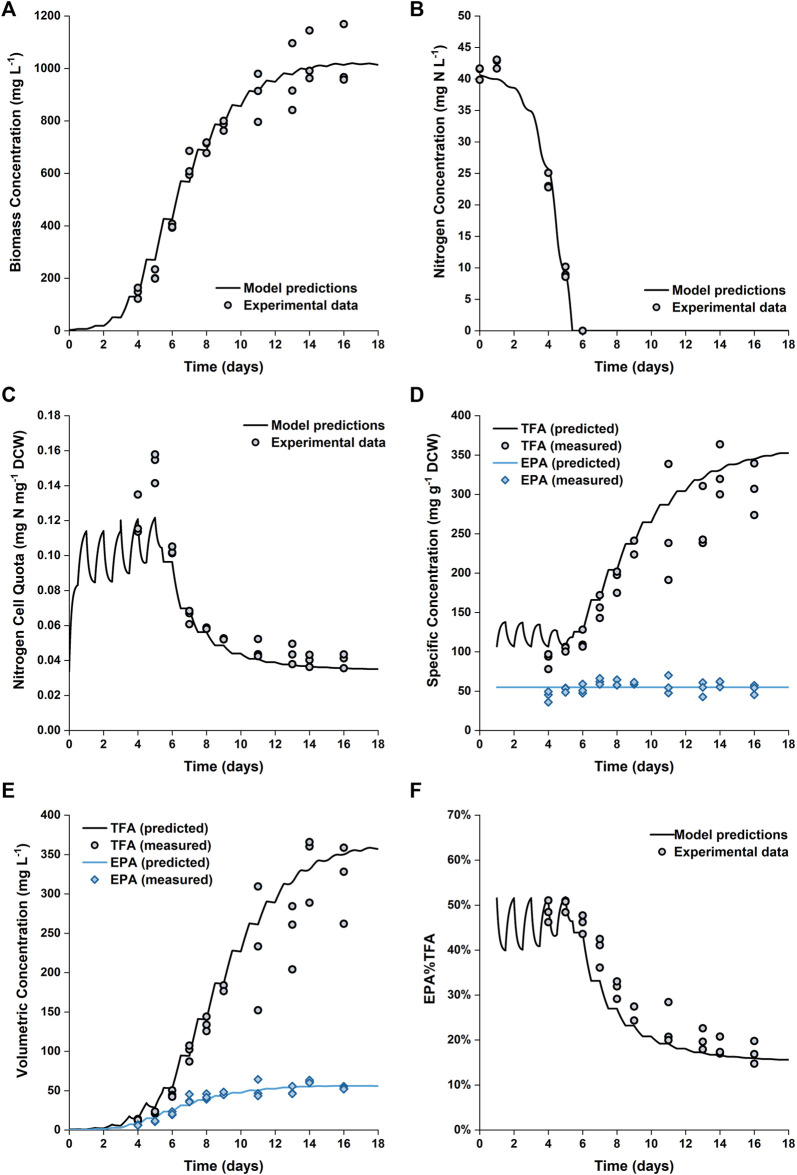
Comparison between experimentally measured and predicted biomass concentration **(A)**, nitrogen concentration in the growth medium **(B)**, intracellular nitrogen cell quota **(C)**, specfic concentrations of total fatty acids and EPA in biomass **(D)**, volumetric concentrations of total fatty acids and EPA **(E)** and EPA fraction in total fatty acids **(F)** of *P. tricornutum* grown in batch cultures in 5 L flat-panel PBRs. Experiments were performed in triplicate with all data points being shown.

Figure 6 shows the comparison between the predicted and experimental results for cultures grown in the 5 L PBRs. The model produces satisfactory predictions for all trends of biomass growth, nitrogen uptake and storage, as well as total fatty acid and EPA production. Nitrogen in the medium was exhausted on Day 6; however, the biomass continued to grow until Day 14. Between Day 6 and Day 14, the internally stored nitrogen supported the biomass to increase by another ∼150% (from ∼400 to ∼1,000 mg L^−1^). This was consistent with the observed change in the nitrogen cell quota, which decreased from ∼0.1 to ∼0.04 mg N mg^−1^ DCW between Day 6 and Day 12. The model predictions of the final biomass concentration and nitrogen cell quota on Days 14–16 are in excellent agreement with the experimental data.

However, the nitrogen cell quota before the onset of nitrogen depletion was slightly underpredicted. The fitted value for $Q_{\max}$ returned by the multivariable multiple regression procedure is 0.12 mg N mg^−1^ DCW; this value was in good agreement with the observed Q on Day 4, but ∼20% lower than the value of Q measured on Day 5 (0.14–0.16 mg N mg^−1^ DCW). For the purposes of comparison the calculated uncertainty in the measurement was of the order 3%, while the variation between replicates was of the order 15–20%. A possible explanation for this difference is ‘surge transport’ of nitrogen which would occur at low external concentrations (i.e., just before depletion in the medium). This would not be captured by the Droop model as the mathematical formulation of the Droop model guarantees that the Q value is bounded between $Q_{\min}$ and $Q_{\max}$ for an initial condition $Q_{\min}\leq Q_{o}\leq Q_{\max}$ (Bernard, 2011). Flynn suggested that an additional parameter, absolute maximum possible quota, could be introduced to account for this surge transport phenomenon under nutrient-sufficient conditions (Flynn, 2008); however, as the underprediction of $Q_{\max}$ does not appear to affect the overall accuracy of the model, such an approach was not adopted in order to keep the complexity of the model structure at a minimal level.

A detailed profile of the fatty acid composition as a function of time is provided in Figure 5. As previously noted, the specific EPA concentration did not vary appreciably with time, while the percentage of EPA as a fraction of the total fatty acids decreased (from ∼50 to ∼17%). This was due to the accumulation of other fatty acids (primarily C16:0 and C16:1), which occurred at the onset of nitrogen limitation. The observed evolutions of TFA and EPA concentrations on both DCW and volumetric bases are well captured by the model, the model also captured the change in the fraction of EPA in the total fatty acids.

### Validation for 50 L PBRs

**FIGURE 7:**
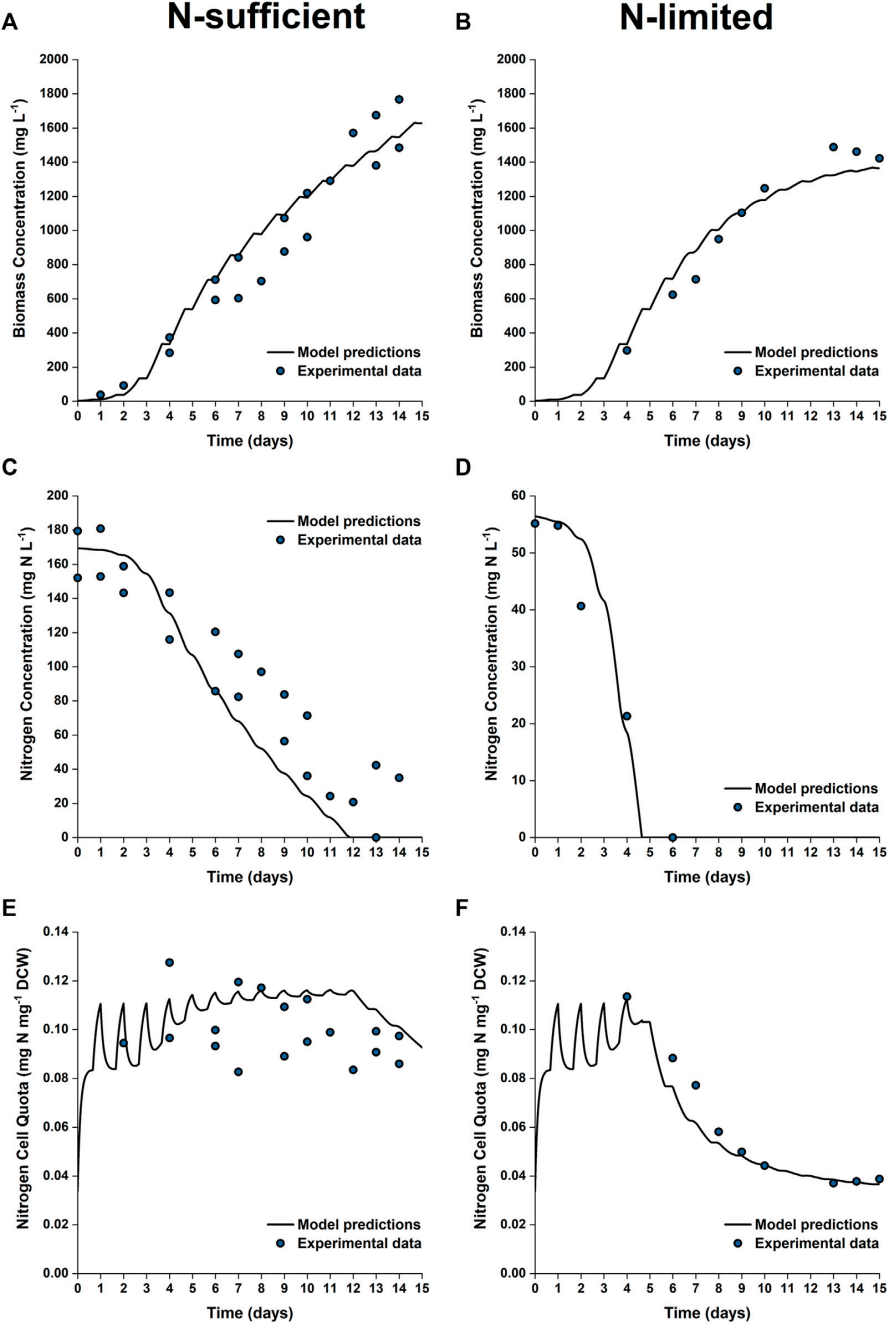
Comparison between experimentally measured and model predicted biomass concentration **(A,B)**, nitrogen concentration in the growth medium **(C,D)** and intracellular nitrogen cell quota **(E,F)** of *P. tricornutum* grown under N-sufficient **(A,C,E)** and N-limited **(B,D,F)** conditions. Batch cultures were grown in 50 L bubble column PBRs, with two biologically independent replicates for N-sufficient conditions and one for N-limited conditions.

To further test the validity of the model at industrially relevant scales, simulations were performed for batch cultures grown in 50 L bubble column PBRs under nitrogen sufficient and nitrogen limited conditions. Figure 7 shows the model predictions plotted against the experimental data for biomass growth and nitrogen uptake. As shown, the model captured the trends of biomass growth under both nutrient conditions, with the model-data mismatch for the final biomass concentration being 5–10%. The observed onset of the stationary phase was in good agreement with the model predictions.

Nitrogen uptake is more accurately predicted under N-limited conditions than N-sufficient conditions. The nitrogen concentration in the medium is slightly underpredicted in the N-sufficient cultures, especially from Day 6 onwards, with the maximum root mean square error between the model prediction and experimental results being 40% between Days 6 and 11. This suggested that nitrogen uptake could be overpredicted under light-sufficient conditions. This discrepancy could be due to the fact that the current model structure is based on the intrinsic assumption that nitrogen uptake is an event independent of biomass growth; nitrogen uptake might be downregulated when there are other factors that limit the growth (Flynn, 2008).

**FIGURE 8:**
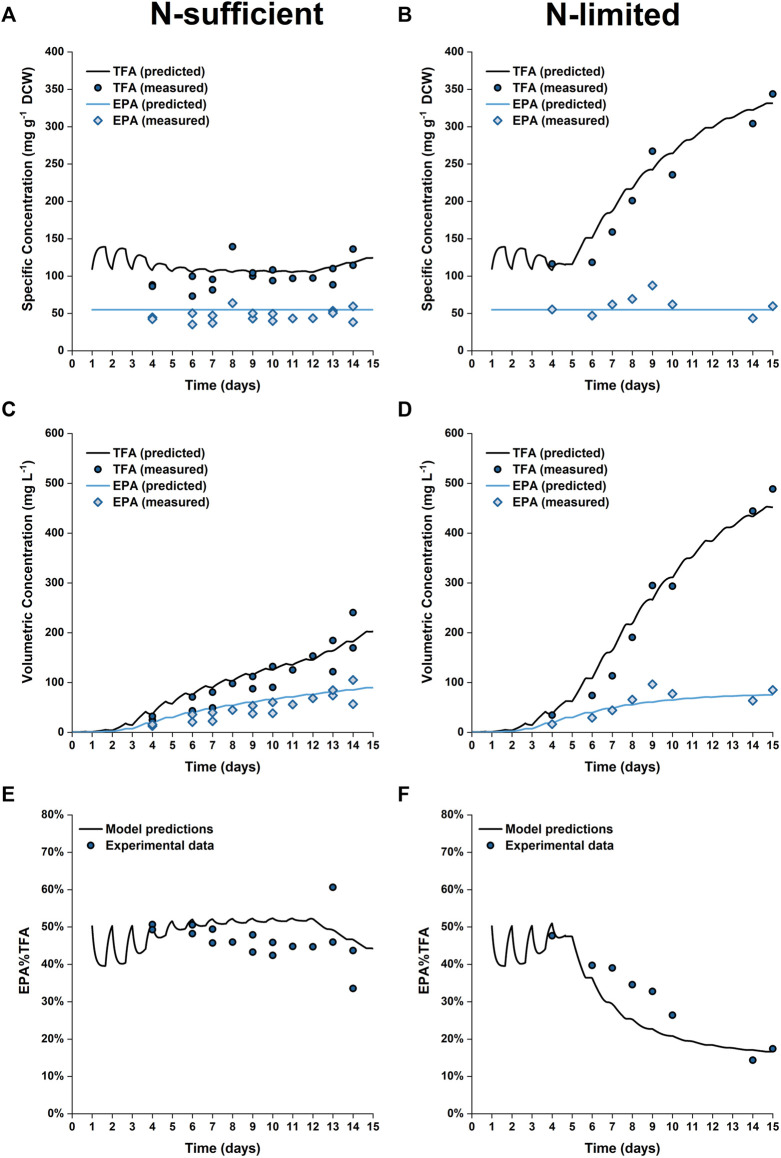
Comparison between experimentally measured and model predicted specific concentrations of total fatty acids (TFA) and EPA in biomass **(A,B)**, volumetric concentrations of TFA and EPA **(C,D)** and EPA fraction in TFA **(E,F)** of *P. tricornutum* grown under N-sufficient **(A,C,E)** and N-limited **(B,D,F)** conditions. Batch cultures were grown in 50 L bubble column PBRs, with two biologically independent replicates for N-sufficient conditions and one for N-limited conditions.

The predicted and experimental results for the EPA and TFA concentrations are in good agreement, as shown in Figure 8. The specific EPA concentrations found in the 50 L bubble column cultures remained relatively stable and were not significantly different to those found in the 5 L flat-panel PBRs (Supplementary Figure S5). Under nitrogen limitation, the specific concentration of EPA remained relatively stable while the specific concentration of TFA increased by ∼200%, similar results were observed in the 5 L scale experiments, as shown in Figure 5. The progressive decrease in the percentage of EPA as a fraction of the total fatty acids from 50 to 17% is well captured by the model; with the model-data mismatch being less than 5%. On the other hand, in the N-sufficient culture there was little change in the specific concentration of TFA and EPA as a percentage of total fatty acids (Figure 5). This is also well captured by the model. The EPA percentage of total fatty acids predicted by the model is slightly higher than the experimental data on Days 6–12, but the model-data mismatch is generally within 20%. This slight overprediction is likely due to the overprediction of the nitrogen cell quota as discussed above.

### Process optimization

The validated model can serve as a useful tool for optimizing EPA production using *P. tricornutum* as well as predicting the product quality (i.e., the EPA concentration in the biomass or the total fatty acids). As previously discussed, since the EPA concentration in the biomass remains approximately constant the objective of any optimization process would be to maximize the biomass productivity. Similarly, if it is desired to produce a lipid extract with a high fraction of EPA the objective would be to both maximize the biomass productivity and avoid nitrogen limitation.

As previously noted the fundamental limitation for photoautotrophic processes is the availability of light. For systems operated in batch mode the biomass productivity will decrease as the batch progresses; this is due to the increase in self-shading at higher cell densities. The volumetric biomass productivities reported in this work (120 mg L^−1^ day^−1^) are less than the maximum values reported in the literature (e.g., 500 mg L^−1^ day^−1^ for a bubble column indoors (Contreras et al., 1998) and 2,760 mg L^−1^ day^−1^ for a tubular reactor outdoors (Fernández et al., 1998)). These differences can be attributed to three factors: 1) the longer path lengths used in this work, 2) the shorter photoperiod used here and 3) the lower light intensities used. To demonstrate this point and as a further check of the model simulations were performed using the conditions of Contreras et al. (1998). It was found that the difference between the predicted and experimentally measured biomass concentration was of the order 5%. This demonstrates the ability for the model to be applied to a wide range of conditions.

**FIGURE 9:**
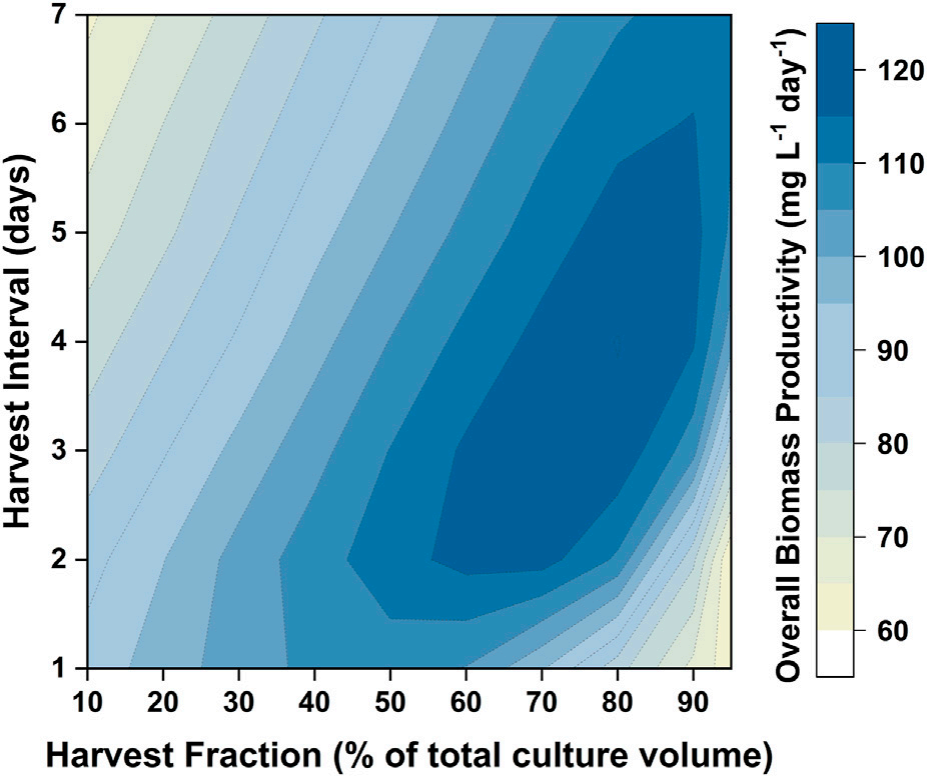
Contour showing model predictions of biomass productivity in repeated-batch cultures of *P. tricornutum* with different harvest intervals and harvest fractions. Simulations were run based on the 5 L flat-panel PBR setup with sufficient nitrogen. The cultures were initially run in the batch mode for 8 days, followed by four havest cycles.

Issues with self-shading can be addressed by changing the reactor design (i.e., reducing the path length) as well as the operating conditions (i.e., increasing the light intensity). Another way in which the productivity can be improved is by changing the operational strategy (i.e., using a repeated batch). In designing such an operating strategy it is necessary to determine the percentage of the medium to be harvested, as well as the interval between these harvests. A major benefit of having a validated model is that it can be used to design the optimum harvesting strategy. For the light intensity examined (350 µmol photons m^−2^ s^−1^) the model predicted that the biomass productivity would reach a maximum after 8 days. Hence, the cultivation strategy tested would consist of an 8 day batch period, followed by repeated cycles where the culture was harvested and replaced by fresh medium. Different combinations of harvest intervals (the time between two harvest cycles) and harvest fractions (the fraction of culture broth to be replaced by fresh medium) were investigated. The results are shown in Figure 9. The highest biomass productivity (115–120 mg L^−1^ day^−1^) over the entire culture duration could be achieved with harvest fractions of 60–80% and harvest intervals of 3–5 days. This optimized biomass productivity is predicted to be 40–50% higher than what can be achieved using a batch mode. Model simulations were also performed to investigate the relationship between the biomass productivity and the number of repeated-batch cycles. It was found that there was minimal increase in the biomass productivity after five repeated cycles (as shown in Supplementary Figure S9). Repeated-batch simulations were also performed for the 50 L bubble column PBRs. The repeated-batch strategy could increase the biomass productivity from approximately 110 mg g^−1^ L^−1^ up to 160 mg L^−1^ day^−1^, consistent with the extent of improvement found in the 5 L flat-panel PBRs.

**FIGURE 10:**
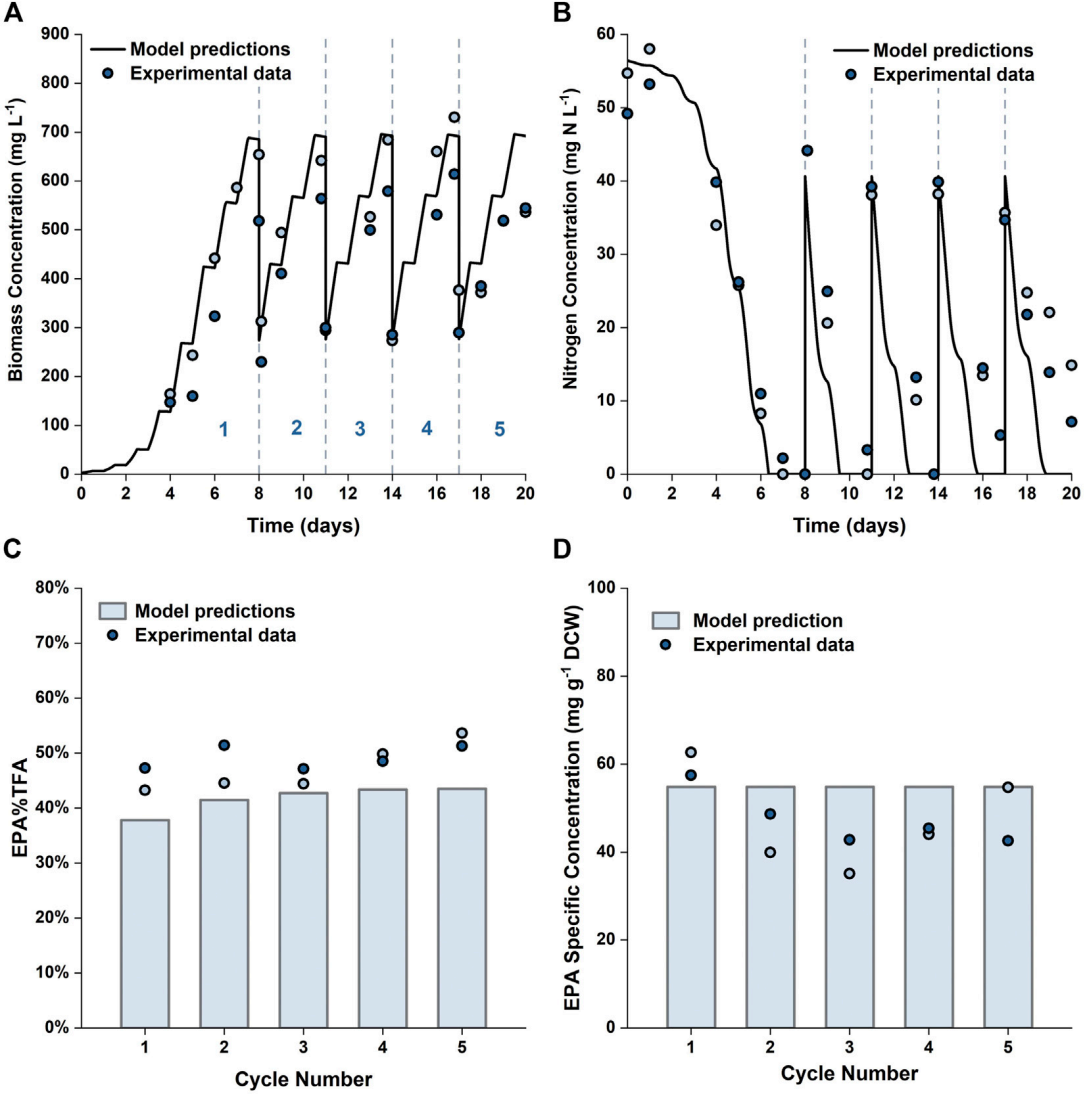
Comparison between experimentally measured and model predicted biomass concentration **(A)**, nitrogen concentration in the growth medium **(B)**, EPA fraction in total fatty acids **(C)** and specific concentration of EPA in biomass **(D)** in repeated-batch cultures of *P. tricornutum* grown in 5 L flat-panel PBRs. The cultures were grown in batch mode in 5 × *f*/2 medium for 8 days, then every 3 days 60% of the culture broth was removed and replaced with 6 × *f*/2 medium. Scatter plots represent two biologically independent replicates.

To further verify the applicability of our model for predicting repeated-batch cultures, validation experiments were carried out in the 5 L flat-panel PBRs where the algae were grown in 5 × *f*/2 medium for 8 days, and then 60% of the culture broth was harvested and replaced with fresh medium prepared at 6 × *f*/2 strength every 3 days. This approach was selected based on the results shown in Figure 9; with the medium composition being chosen to avoid nitrogen limitation. A consequence of this was that the specific EPA concentration and the percentage of EPA as a fraction of the total fatty acids remained approximately constant throughout the repeated batch, a factor which is advantageous from a product quality perspective. The experimental results are plotted against the model simulations in Figure 10. It was possible to operate the cultures in the repeated-batch mode for at least four harvest cycles. During the fifth cycle a decline in biomass productivity was observed in both replicates. A white biofilm was observed to form on the reactor surface, suggesting that the reduced viability could be caused by microbial contamination. As the experiments were performed without sterilizing the growth medium or the reactor, it is not surprising that there was some contamination, especially as the duration of the batch increased. As running the cultures beyond four repeated-batch cycles would not further improve the productivity significantly (as shown in Supplementary Figure S9), from an economic point of view, it is may not be worthwhile to eliminate potential sources of contamination in order to increase the number of cycles.

The model predictions were in reasonable agreements with the experimental results. Compared with batch cultures run for an equal duration, the overall biomass and EPA productivities were improved by 45–56% and 6–29%, respectively. These results are in line with the model predictions and clearly demonstrate the usefulness of the model in terms of process development and optimization.

An obvious avenue for future work would be to use the model as a tool in the techno-economic analysis of large-scale EPA production. Future work could also extend the model to include more products (e.g., pigments, proteins). Such models would serve as a good starting point for the techno-economic analysis of co-production of microalgal EPA and other bioproducts, which presents a potential avenue for improving the overall process economics. Due to the relatively stable EPA content in *P. tricornutum* biomass, the EPA productivity could be improved by increasing the biomass productivity using strategies that avoid severe light and nutrient limitation (e.g., repeated-batch cultivation). However, production of other compounds may be more sensitive to culture conditions. For example, the accumulation of fucoxanthin is favored by low light conditions (McClure et al., 2018). Therefore, the optimal conditions for the co-production process might be different to those for a single product. In this instance the modelling approach may offer significant advantages as it offers a way of determining the growth conditions which maximize the productivity of multiple products.

## Conclusion

This study set out to develop and validate a kinetic model capable of predicting the biomass, total fatty acid and EPA production of *P. tricornutum*. The effects of light and nitrogen availability were accounted for using Monod and Droop models, respectively, while the TFA and EPA concentrations were modelled using empirical correlations based on the nitrogen cell quota. The proposed model was mathematically simple yet provided satisfactory predictions for a range of different culture conditions. These included different reactor designs, scales, and operating conditions. The modelling approach used in this work could be readily extended to other reactor designs and operating conditions, provided suitable information (i.e., geometric data and light intensities) were available. Similarly, the modelling framework could also be used to model the growth and EPA production of other species of microalgae, provided information about key parameters (e.g., the specific growth rate and nitrogen cell quota) was known.

The model developed in this work can be also used for process optimization. For example, as previously described it was predicted that the use of an optimal repeated-batch strategy would lead to an approximately 50% increase in the biomass productivity, with this prediction being validated by experimental work. Given that the model has been shown to successfully predict the biomass and EPA productivities for a range of conditions it can clearly serve as a useful tool in the design, optimization and scale-up of processes for the microalgal production of EPA.

## Data Availability

The data underpinning this publication can be accessed from Brunel University London's data repository, Brunelfigshare here under a CCBY licence: https://doi.org/10.17633/rd.brunel.21197263.v1.

## Glossary

$ABS_{220}$: Absorbance at 220 nm (−)
$ABS_{275}$: Absorbance at 275 nm (−)
$ABS_{550}$: Absorbance at 550 nm (−)
$C_{TFA}$: Specific concentration of total fatty acids (mg g^−1^ DCW)
DCW: Dry cell weight (mg L^−1^)
DF: Dilution factor (−)
I: Local light intensity at a given point in a photobioreactor (μmol m^−2^ s^−1^)
$I_{av}$: Average light intensity in the culture (μmol m^−2^ s^−1^)
$I_{o}$: Incident light intensity (μmol m^−2^ s^−1^)
$K_{a}$: Biomass absorption coefficient (L mg^−1^ m^−1^)
$K_{d}$: Biomass decay rate (day^−1^)
$K_{I}$: Half-saturation constant of light (μmol m^−2^ s^−1^)
$K_{N}$: Half-saturation constant of nitrogen (mg N L^−1^)
L: Depth of 5 L flat-panel PBRs (m)
l: Distance between the illuminated surface a given point in a photobioreactor
m: Number of biologically independent replicates used for the multiple multivariate regression analysis
n: Exponent (−)
N: Nitrogen concentration in the growth medium (mg N L^−1^)
$N_{o}$: Initial concentration of nitrogen in the growth medium (mg N L^−1^)
NRMSE: Normalized root-mean square error
p: Number of state variables used in the multiple multivariate regression analysis
Q: Nitrogen cell quota (mg N mg^−1^ DCW)
q: Number of days where samples were collected for the multiple multivariable regression analysis
$Q_{\min}$: Minimum nitrogen cell quota (mg N mg^−1^ DCW)
$Q_{\max}$: Maximum nitrogen cell quota (mg N mg^−1^ DCW)
R: Radius of 50 L bubble column PBRs (m)
r: Radial coordinate of a given point in polar coordinates (m)
U: Nitrogen uptake rate (mg N mg^−1^ DCW day^−1^)
$U_{\max}$: Maximum nitrogen uptake rate (mg N mg^−1^ DCW day^−1^)
w: Weighting factor
X: Biomass concentration (mg L^−1^)
Y: Predicted value of a state variable
$\hat{Y}$: Observed value of a state variable
θ: Angular coordinate of a given point in polar coordinates (radians)
μ: Specific growth rate (day^−1^)
$\mu_{\max}$: Maximum growth rate (day^−1^)
