# Supplementary material for: A scalable model for EPA and fatty acid production by Phaeodactylum tricornutum
Source: Front Bioeng Biotechnol. 2022 Oct 12;10:1011570. doi: 10.3389/fbioe.2022.1011570 (PMC9597327; doi:10.3389/fbioe.2022.1011570)
Supplement: Supplementary file 1 [file Table1.DOCX]

Supplementary Material

# Supplementary Figures and Tables

**Supplementary Figure 1.** Light profile of the 5 L flat-panel PBRs. Measurements were made on the front face of the PBR, with four 9W light strips attached to the back face. The measurements were performed with the PBR filled with cell-free medium and at an air flow rate of 5 L min^-1^.


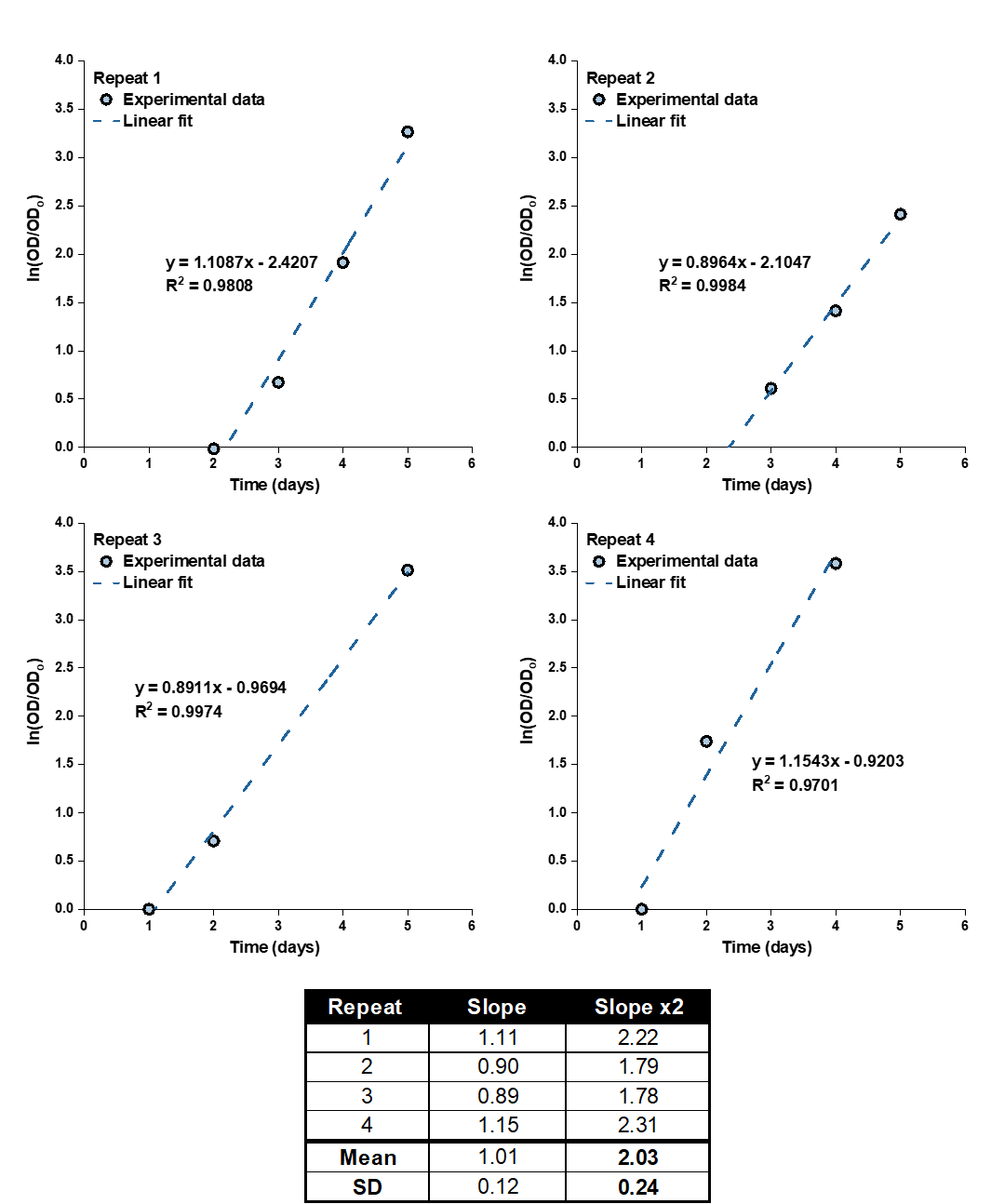


**Supplementary Figure 2.** Estimates of the initial value of $\mu_{max}$ for the multivariable multiple regression procedure.

**Supplementary Figure 3.** Distribution of specific concentration of EPA in *P. tricornutum* biomass grown in 5 L flat-panel PBRs (*N* = 30). Normality was checked using the Kolmogorov-Smirnov test and the Lilliefors test in OriginPro 2019b, which returned *p* = 1 and *p* = 0.2, respectively. Estimates of the initial value of $\mu_{max}$ for the multivariable multiple regression procedure.

**Supplementary Figure 4.** Distribution of specific concentration of EPA in *P. tricornutum* biomass grown in the 50 L bubble column PBRs. Normality was checked using the Kolmogorov-Smirnov test and the Lilliefors test in OriginPro 2019b, which returned *p* = 0.42 and *p* = 0.063, respectively. A two-tail t-Test was performed in OriginPro 2019b to compare the specific EPA concentrations in the 50 L bubble column PBRs and those in the 5 L flat-panel PBRs (shown in Figure S4): the two sets of data were not significantly different at α = 0.05.

**Supplementary Figure 5.** Effect of ±20% variation in each of the model parameters on the predicted biomass concentration under nitrogen-sufficient conditions.

**Supplementary Figure 6.** Effect of ±20% variation in each of the model parameters on the predicted nitrogen cell quota under nitrogen-limited conditions.

**Supplementary Figure 7.** Effect of ±20% variation in each of the model parameters on the predicted biomass concentration under nitrogen-limited conditions.

**Supplementary Figure 8.** Effect of ±20% variation in each of the model parameters on the predicted nitrogen cell quota under nitrogen-limited conditions.

**Supplementary Figure 9.** Model predictions of the biomass productivity as a function of number of repeated-batch cycles. Simulations were run based on the 5 L flat-panel PBR setup with sufficient nitrogen. The cultures were initially run in the batch mode for 8 days, after which 60% of the culture was harvested and replaced by fresh medium every three days.
